# Tau Isoform-Driven CBD Pathology Transmission in Oligodendrocytes in Humanized Tau Mice

**DOI:** 10.3389/fneur.2020.589471

**Published:** 2021-01-15

**Authors:** Justyna Zareba-Paslawska, Kalicharan Patra, Luca Kluzer, Tamas Revesz, Per Svenningsson

**Affiliations:** ^1^Laboratory of Translational Neuropharmacology, Department of Clinical Neuroscience, Karolinska Institute, Stockholm, Sweden; ^2^Queen Square Brain Bank, Department of Clinical and Movement Neurosciences, Queen Square Institute of Neurology, University College London, London, United Kingdom

**Keywords:** tau, CBD, oligodendrocytes, myelin basic protein, hTau mice

## Abstract

The aggregation of abnormally phosphorylated tau protein in neurons and glia is a neuropathological hallmark of several neurodegenerative disorders, collectively known as tauopathies. They are further subclassified based on the preferential pathological aggregation of three carboxyl-terminal repeat domains (3R) and/or 4R tau. Corticobasal degeneration (CBD) is a rare neurodegenerative disorder classified as a 4R tauopathy. In the present study, we extend analysis of CBD-tau cell-type specific pathology transmission with 3R and 4R tau isoform distinguishable changes. We use a humanized tau (hTau) mouse line, which overexpress all six human tau isoforms in a murine tau knockout background and perform intrastriatal inoculation of control and CBD-tau enriched human brain homogenate. We show that CBD-tau causes hyperphosphorylation of tau at Ser202 predominantly in oligodendrocytes. Next, we demonstrate the spread of tau pathology from striatum to the overlaying corpus callosum and further to the contralateral side. Finally, we demonstrate that the almost exclusive oligodendrocyte-based transmission of hyperphosphorylated tau is reflected in the endogenous 4R tau isoform expression and corresponds to subclassification of CBD as a 4R tauopathy. Additionally, we identify functional changes in oligodendrocytes reflected by myelin basic protein abnormalities upon CBD-tau inoculation. These changes are not observed in murine tau knockout mice lacking both human and murine tau. Our study presents not only *in vivo* tau isoform–driven region- and cell-specific tau pathology, but also underlines that tau pathology seeding and transmission might be oligodendrocyte-based. These results, which need to be extended to more cases, give new insights into why tauopathies might vary greatly in both histopathological and neuroanatomical patterns.

## Introduction

Corticobasal degeneration (CBD) is a rare neurodegenerative disorder with 0.6–0.9 cases per 100,000 individuals per year (1, 2), and in a more recent study of a Russian population, it was estimated at 0.02 cases per 100,000 individuals per year (3). The cardinal CBD features are progressive asymmetric rigidity and apraxia, accompanied by aphasia and dystonia. The neuropathological hallmark of CBD is astrocytic plaque formation and accumulation of abnormally phosphorylated tau in neurons and glia (oligodendroglia and astrocytes) predominantly in forebrain structures (4). Although the CBD tau pathology is mainly astrocytic and neuronal, the oligodendroglia tau aggregates are also present in a form of numerous and widespread cytoplasmic process inclusions (argyrophilic threads) (5) and oligodendroglia cell body inclusions (coiled bodies) (6). Although astrogliopathy predominates in the earliest stage of CBD pathology, the oligodendroglia involvement in the early preclinical stages is also reported (7). Furthermore, in connection to the oligodendroglia pathology, the white matter volume loss in subcortical structure is observed in CBD (8, 9). Although CBD is a predominantly sporadic disorder, familial cases with tau protein mutation (N296N) have been reported (10).

Abnormalities in tau protein, resulting in its aggregation in neurons and glia, are a neuropathological hallmark of more than 20 neurodegenerative disorders collectively named tauopathies (11). Human tau protein is encoded by the microtubule associated protein tau (MAPT) gene containing 16 exons. The alternative splicing of exon 2 (E2), E3, and E10 result in expression of 6 tau isoforms in the adult human brain. The E2 and E3 determine the presence of 0, 1, or 2 near-amino-terminal inserts (0N, 1N, or 2N, respectively), and the existence of 3 or 4 carboxy-terminal repeat domains (3R or 4R, respectively) is dictated by alternative splicing of E10, in which only 4R variants include E10. The carboxy-terminal repeat domains are part of the microtubule assembly domain, and the presence of an extra repeat in 4R tau accelerates the microtubule assembly process 2.5–3.0 times compared to 3R (12). The presence or absence of E10 is particularly interesting in tauopathies. The preferential accumulation of 3R and/or 4R tau provides a subclassification of tauopathies (13). The most frequent neurodegenerative disorder, Alzheimer disease (AD), is a 3R + 4R tauopathy; progressive supranuclear palsy (PSP), CBD, and argyrophilic grain disease are 4R tauopathies; and Pick's disease (PiD) represents a 3R tauopathy (13). The tau protein also undergoes many post-translational modifications, among which phosphorylation is of particular interest due to insoluble hyperphosphorylated tau deposits found in postmortem brains from patients with tauopathy (14). Since the first classification of tau as a phospho-protein (15), at least 85 phosphorylation sites have been identified (16). Under normal physiological conditions, phosphorylation/dephosphorylation of tau modulates its microtubule binding properties. Tau affinity to the microtubule decreases upon phosphorylation (14), which increases the amount of more aggregation-prone cytosolic tau fraction (17). In addition, a recently published study challenges the current hypothesis that tau protein is a microtubule-stabilizing protein in axon and favor a new theory in which tau rather enables axonal microtubules to have the long labile domains (18).

Over the years, much attention has been placed on the neuronal connectome from the injection side and specific tau strains to elucidate the intraneuronal spread of tau aggregates in tauopathies. Recently, more focus is placed on explaining the role of astrocytes, oligodendrocytes, and microglia in tau-driven neurodegenerative disorders. Moreover, the striatum and prefrontal cortex are indicated as the earliest sites of tau pathology in CBD (7). Therefore, we utilize humanized tau (hTau) animals, overexpressing all six human tau isoforms in a murine knockout background and perform intrastriatal CBD-tau enriched brain homogenate inoculation to study a CBD-tau cell-type-specific pathology transmission. We show an oligodendrocyte predominant disease transmission, which we further identify to be 4R tau dependent.

## Materials and Methods

### Subjects and Sample Collections

Subjects' consent was obtained according to the Declaration of Helsinki. All experiments involving human subjects were approved by the regional ethical committees at Karolinska University Hospital (2016/19–31/1). Formalin-fixed, paraffin-embedded 5-μm human brain sections and fresh frozen human brain blocks from subjects without neurological disorders (*n* = 1) or pathologically typical CBD (*n* = 1) were obtained from the Queens Square Brain Bank, London. The control case was an 88-year-old male with pathological aging-related amyloid beta deposition, limbic TDB-43, and mild small vessel disease. The CBD case was a 78-year-old male with characteristic CBD pathology, including typical AT8 positive astrocytic plaques, neuropil threads, and scattered oligodendroglial coiled bodies in the subcortical white matter. He also exhibited pathological aging (CERAD sparse amyloid beta plaques), Braak & Braak stage II. A detailed neuropathological description of the cases is presented in the Supplementary Data.

### Purification of Insoluble Tau From CBD and Control Brains

The fresh frozen blocks of human frontal cortex from the CBD and control cases were homogenized at 1:5 (w/v) in sterile phosphate-buffered saline (PBS) with complete protease inhibitors (1836153, Roche, Basel, Switzerland), sonicated 5 × 1 s and centrifuged at 3,000 *g* at 4°C for 5 min. Next, the supernatant was centrifuged at 100,000 *g* at 4°C for 20 min. The supernatant, containing soluble tau, was aliquoted and stored at −70°C, and the pellet fraction containing insoluble tau was resuspended in the original volume of homogenization buffer, sonicated 5 × 1 s, aliquoted, and stored at −70°C (19).

### Animals

hTau transgenic mice B6.Cg-Mapt^tm1(EGFP)Klt^ Tg(MAPT)8cPdav/J (stock #004808 005491, The Jackson laboratory, Bar Harbor, ME, USA) and tau knockout (KO) mice B6.Cg-Mapt^tm1(EGFP)Klt^ (stock #004779, The Jackson laboratory, Bar Harbor, ME, USA) were bred in the laboratory according to supplier instructions.

### Stereotaxic Surgery

The 3-month-old mice were randomly assigned (4–8 per group) to the experimental groups. All experiments were performed in agreement with the European Communities Council (86/609/EEC) and approved by the Stockholm North Ethical Committee (Ethical permit # N13014). Stereotaxic surgery was performed under anesthesia in a stereotaxic frame (Stoelting, Wood Dale, IL, USA). The right striatum was injected with 8 μg of insoluble tau fractions from brain lysates of CBD or control cases at the coordinates 0.8 mm posterior, 1.95 mm lateral to bregma, and 3.0 mm ventral according to bregma. The infusion of 2 μl was conducted with a 10-μl Hamilton syringe (Hamilton, Bonaduz, Switzerland) at a rate of 0.2 μl/30 s. After infusion completion, the needle was left at the position for an additional 5 min and then slowly retracted. The skin was sutured and postoperative pain relief, Temgesic (s.c.; Indivior UK Limited, Slough, UK), was given directly after the surgery and additionally two times within 48 h. The animals were placed back in the cage with unlimited access to water and food until the experimental endpoint.

### Immunocytochemistry

At 1 and 12 months postsurgery, mice were deeply anesthetized and perfused transcardially with saline followed by buffered 4% paraformaldehyde. Both hemispheres were collected, dehydrated, and embedded in paraffin and then sectioned into 4-μm-thick sagittal sections using a microtome. Next, slides were deparaffinized, rehydrated, and subjected to the antigen retrieval step in sodium citrate pH 8.5 for 30 min at 80°C. All washes were made with 1X PBS (7.2 mM Na_2_HPO_4_, 2.8 mM NaH_2_PO_4_, 140 mM NaCl, pH 7.4). Blocking of endogenous IgG with a Mouse on Mouse (M.O.M.™) Basic kit (BMK-2202, Vector Laboratories, Burlingame, CA, USA) was performed according to manufacturer protocol for mouse primary antibodies. The overnight incubation was performed at 4°C with the following primary antibodies: CP13 antibody (gift from P. Davis, dilution 1:1,000) for detection of phosphorylated tau at Ser202, Olig2 (ab109186, Abcam, Cambridge, UK; dilution 1:300), Sox9 (ab185966, Abcam, Cambridge, UK; dilution 1:300), NeuN (ab177487, Abcam, Cambridge, UK; dilution 1:300), MBP (sc-271524, Santa Cruz Biotechnology, Dallas, TX, USA; dilution 1:300), and RD4 (05-804, Merck Millipore, Burlington, MA, USA; dilution 1:300). Next, sections were washed and quenched of endogenous peroxidase in 3% hydrogen peroxide and 10% methanol in PBS. Thereafter, sections were washed, and secondary antibody incubation was performed with M.O.M.™ Biotinylated Anti-Mouse IgG Reagent (BMK-2202, Vector Laboratories, Burlingame, CA, USA) or ImmPRESS™-AP REAGENT Anti-Rabbit IgG (MP-5401, Vector Laboratories, Burlingame, CA, USA) according to manufacturer protocol. The subsequent washes were performed, and avidin-biotin complex horseradish peroxidase kit (ABC Elite) (PK-6100, Vector Laboratories, Burlingame, CA, USA) with 3,3-diaminobenzidine (DAB, D5637, Sigma-Aldrich, St. Louis, MO, USA) based detection, yielding brown product, was applied for CP13, MBP, and RD4 antibody detection. The Vector® Red AP substrate (SK-5100, Vector Laboratories, Burlingame, CA, USA), yielding red/pink reaction product, was applied for Olig2, Sox9, and NeuN antibodies. The multiple antigen labeling was performed for detection of CP13 or RD4 antibody with DAB chromogen and followed by subsequent Olig2, Sox9, or NeuN overnight incubation and detection with Vector® Red AP substrate. Negative controls, omitting primary antibodies, were always conducted in parallel for immunohistochemical staining. The human paraffin sections were treated as described above for mice sections with the following alterations: no M.O.M. kit incubation was applied, and horseradish peroxidase directly conjugated secondary antibody (P044701-2, Dako, Denmark; dilution 1:100) was applied to detect CP13 mouse antibody.

### Sodium Dodecyl Sulfate Polyacrylamide Gel Electrophoresis (SDS-PAGE)

The SDS-PAGE was performed as previously described (20). The sample was mixed in a 3:1 ratio with denaturing loading buffer (106 mM Tris-HCl, 141 mM Tris, 2% lithium dodecyl sulfate (LDS), 10% glycerol, 6% β-mercaptoethanol, 0.51 mM EDTA, 0.22 mM SERVA Blue G-250, 0.175 mM Phenol Red, pH 8.5) and boiled at 95°C for 5 min. Next, samples were separated using a bis-tris acrylamide gel using MES running buffer (50 mM Tris, 50 mM 2-(N-morpholino) ethanesulfonic acid (MES), 0.1% SDS, 1 mM EDTA, pH 7.3).

### Western Blotting

After SDS/PAGE, gels were assembled with 0.45 μm pore size polyvinylidene difluoride membranes, and semidry transfer was performed. Membranes were blocked during 1 h incubation in 5% skim milk at RT. Next, membranes were incubated overnight at 4°C with a primary antibody diluted 1:1,000 (vol/vol) in 1% skim milk. Membranes were thereafter incubated for 2 h with appropriate horseradish peroxidase (HRP)-conjugated secondary antibody (Dako, Denamrk) diluted 1:10,000 (vol/vol) in 1% skim milk at RT. The signal was developed by a Clarity Western ECL Substrate (BioRad, USA). A colorimetric scan was used to visualize the protein ladder. The intensity of protein bands was analyzed using ImageJ (21). Primary antibodies used were RD3 (05-803, Merck Millipore, USA), RD4 (05-804, Merck Millipore, USA), CP13 (gift from P. Davis, 1:5,000 dilution), AT8 (MN1020, Thermo Fisher Scientific, USA), AT100 (MN1060, Thermo Fisher Scientific, USA), pThr181 (12855, Cell Signaling, USA) and DA9 (gift from P. Davis).

### Dot Blotting and Ponceau S Staining

One μl of the insoluble tau fractions from brain lysates of CBD and control cases was placed on a nitrocellulose membrane and air dried. The Ponceau S staining (P7170-1L, Sigma-Aldrich, St. Louis, MO, USA) was performed according to manufacture protocol. A colorimetric scan was used to visualize the signal. The intensity of the protein signal was analyzed using ImageJ (21).

### Negative Stain TEM

Three μl of the sample was applied on glow-discharged, carbon-coated, and formvar-stabilized 400 mesh copper grids (Ted Pella) and incubated for approximately 30 s. Excess sample was blotted off, and the grid was washed with MilliQ water prior to negative staining using 2% uranyl acetate. TEM imaging was done using Hitachi HT7700 (Hitachi High-Technologies) transmission electron microscope operated at 100 kV equipped with a 2k x 2k Veleta CCD camera (Olympus Soft Imaging System).

### Cell Counting

Cell counting was performed on representative light microscopy pictures. The digital images of the striatum and corpus callosum were obtained by a microscopic objective lens (20×, Plan Fluor; Nikon, Tokyo, Japan) and analyzed using ImageJ. For each animal, three sections were analyzed. From each section, three locations of the structures were selected for microphotography. For the striatum, the two dorsal (one anterior and one posterior) and one ventral images were analyzed. The dorsal anterior striatum analysis was performed, omitting the internal capsule. For the corpus callosum, pictures corresponding to the anterior, medial, and posterior parts of the corpus callosum overlaying the striatum were analyzed. The manual exhausted counting of single and double positive cells was performed blindly. The data are presented as a fraction of double positive (CP13^+^/Olig2^+^) cells of all single positive (Olig2^+^) cells and displayed as a percentage of control injected animals.

### Densitometry

The optical density of MBP immunoreactivity was performed on representative light microscopy pictures. The digital images of the corpus callosum were obtained by a microscopic objective lens (10×, Plan Fluor; Nikon, Tokyo, Japan) during one microscopic session and analyzed in gray scale using ImageJ. The same microscope light intensity was applied to all acquired pictures. For each animal, three sections were analyzed. From each section, three locations from the anterior, medial, and posterior parts of the corpus callosum overlaying the striatum were selected, omitting backgrounds gaps. The picture collections and data analysis were performed blindly. The optical density for each location was corrected for non-specific background density. The data are presented as a percentage of control injected animals.

### Statistical Analysis

Statistical analyses were performed using GraphPad Prism software (GraphPad Inc). A Mann–Whitney *U* test was applied for nonparametric comparison between two groups. The Student *t* test was applied for parametric comparison between two groups after assessing normality with the Shapiro–Wilk normality test. A Spearman's rank correlation coefficient was used to analyze dependence between two sets of data. All statistical tests were 2-tailed, and a *P*-value < 0.05 was considered statistically significant.

## Results

To investigate cell-specific propagation of CBD-tau, we prepared an insoluble tau fraction from a neuropathologically confirmed CBD case and a control non-neuropathological case. The detailed neuropathological case description is presented in the Supplementary Data. The Western blot analysis of the tau insoluble fraction of control and the CBD brain homogenate used for inoculation is presented in Supplementary Figure 1A. The 3R tau isoforms are presented in both CBD and control cases, and no high molecular weight aggregates were detected in either of them. Importantly, the 4R tau isoforms were also detected in both cases, but only the CBD case possessed a high molecular aggregated form of 4R tau. Further analysis reveals that only the CBD case possessed CP13 (pSer202), AT8 (pSer202, pThr205), AT100 (pThr212, pSer214), and pThr181 positive tau in the tau insoluble brain homogenate fraction. All of them were also detected as a high molecular weight species. In addition, the CBD inoculated material had 3-fold greater signal intensity for total tau than the control case (DA9, pan-tau antibody). Importantly, Ponceau S staining showed that the protein load was 2-fold lower in the CBD case than in the control. Therefore, the observed greater total tau load in the CBD case was due to tau pathology, not difference in total protein load. Further, we performed TEM analysis of injected insoluble material from both the CBD and control cases. Only a few short tangles were observed in the control case (Supplementary Figure 1B). In material isolated from the CBD case, numerous short tangles can be seen (Supplementary Figure 1C). We have not observed any long tangles in any of the samples, which is connected with performing a sonication step of the material prior to injection into mice brains. In addition, we performed immunohistochemistry analysis of frontal cortices from the control and CBD cases using CP13 antibody (Supplementary Figures 1D,F) specific for tau phosphorylated at Ser202 (pSer202), commonly used to detect tau pathology in both early (pretangle) and more advanced stages of neurofibrillary tangle accumulation (22–24). The CP13 positive (CP13^+^) astrocytic plaques were observed only in the CBD case. The neuronal CP13 staining was regularly detected in the CBD case, and only few neurons were observed in the control case. The aging-related tau astrogliopathy was present in both cases in the form of CP13^+^ thorn-shaped astrocytes. No fuzzy CP13^+^ astrocytes were detected in any of the cases. The negative microphotography representation of CP13 staining highlights abundant pSer202 involvement in CBD pathology compared to the control case (Supplementary Figures 1E,G).

The insoluble tau fraction of CBD or control case brain homogenate was stereotaxically injected into the right striatum of 3-month-old humanized tau (hTau) animals and tau knockout mice (KO) based on neuropathological findings highlighting the striatum in preclinical and early stages of CBD (7). The hTau mice overexpress all six human tau isoforms under the tau promoter in the murine tau KO background. Next, we examined tau pathology at 1 and 12 months post-surgery using CP13 antibody (pSer202, pretangles, and more advanced stages of neurofibrillary tangle accumulation) (22–24).

**Figure 1:**
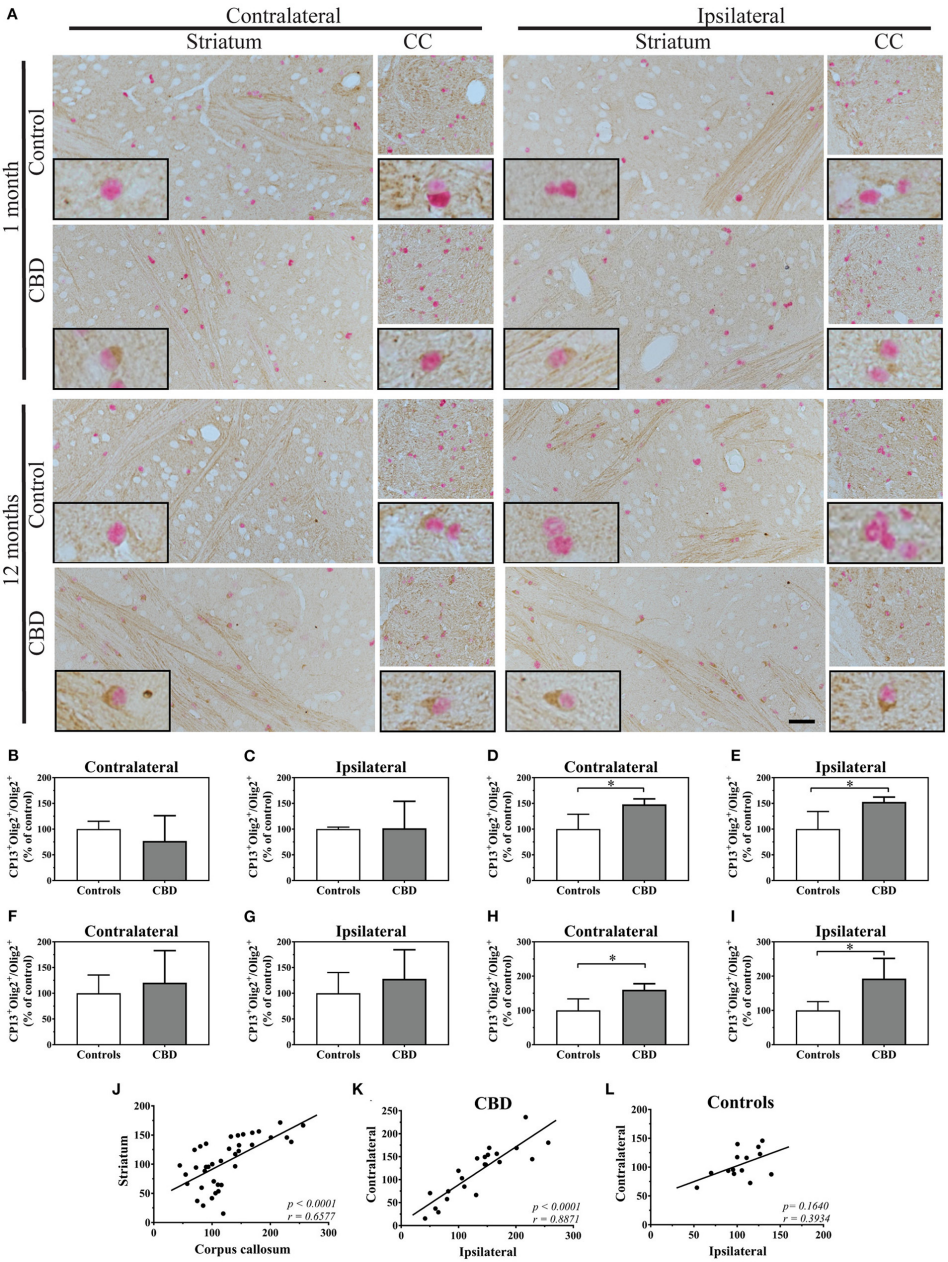
The tau pathology transmission in oligodendrocytes. **(A)** The microphotography pictures of double immunohistochemistry staining of tau phosphorylated at Ser202 (CP13 antibody, brown chromogen) in oligodendrocytes (Olig2, nuclear marker, red/pink chromogen). The cell distribution in the contralateral (left panel) and ipsilateral side (right panel) of the striatum (left subpanel) and corpus callosum (right subpanel) after 1 month (upper panel) and 12 months (lower panel) of control-tau (upper subpanel) or CBD-tau (lower subpanel) inoculation. The representative pictures of cells in each group are presented as the black bordered inserts. The presence of pathological tau in oligodendrocytes increases upon CBD-tau inoculation at 12 months postsurgery. No changes for control-tau were observed. The scale bar represents 50 μm and applies to all pictures; the black-bordered inserts represent 4× digital enlargement. **(B–I)** the quantitative analysis of double positive oligodendrocytes (CP13^+^/Olig2^+^): 1 month postsurgery in the contralateral (**B**, controls *n* = 3, CBD *n* = 8) and ipsilateral (**C**, controls *n* = 4, CBD *n* = 8) striatum; 12 month postsurgery in the contralateral (**D**, controls *n* = 6, CBD *n* = 4) and ipsilateral (**E**, controls *n* = 4, CBD *n* = 4) striatum; 11 months postsurgery in the contralateral (**F**, controls *n* = 3, CBD *n* = 7) and ipsilateral (**G**, controls *n* = 4, CBD *n* = 7) corpus callosum; and 12 month postsurgery in the contralateral (**H**, controls *n* = 5, CBD *n* = 4) and ipsilateral (**I**, controls *n* = 4, CBD *n* = 4) corpus callosum. The significant increase of tau phosphorylated at Ser202 positive oligodendrocytes in CBD-tau injected animals was observed in striatum and corpus callosum 12 months postsurgery compared to control-tau animals. **(J–L)** the correlation of double-positive oligodendrocytes (CP13^+^/Olig2^+^) as a fraction of all oligodendrocytes between the corpus callosum and striatum **(J)**, between the ips- and contralateral side of CBD-tau **(K)** and control-tau **(L)** injected animals. The striatum and corpus callosum transmissible changes in oligodendrcytic tau pathology were detected by the presence of the significant positive correlation between them **(J)**. The significant positive correlation between ipsi-and contralateral hemispheres of oligodendrcytic tau pathology was observed only in CBD-tau **(K)**, and not control-tau **(L)** injected animals strengthening that only pathological tau is able to trigger transmissible changes in oligodendrocytes across hemispheres. Comparison between 2 groups was performed using the Mann–Whitney *U* test. Spearman's rank correlation coefficient was used to analyze dependence between 2 sets of data. *P* value < 0.05 was considered statistically significant. *P < 0.05.

We started analysis of oligodendrocytes using the Olig2 nuclear marker combined with CP13 immunoreactivity. Interestingly, 1 month after inoculation, the vast majority of CP13 positive (CP13^+^) oligodendrocytes in the striatum were located within or in close proximity to white matter tracts, and CP13 negative (CP13^−^) were equally distributed (Figure 1A). No significant differences were observed in Ser202 phosporylated tau positive oligodendrocytes (CP13^+^/Olig2^+^) between control-tau and CBD-tau injected hTau animals in either contra- or ipsilateral striatum (Figures 1B,C, respectively). However, 12-month inoculation of CBD-tau resulted in a significantly higher presence of CP13^+^ oligodendrocytes in the striatum (Figures 1A,D,E). Interestingly, the location of the CP13^+^ oligodendrocytes was no longer mainly restricted to the white matter tracts and their near proximity, but CP13^+^ oligodendrocytes were also regularly observed in striatal gray matter (Figure 1A). Importantly, the significantly increased number of CP13^+^ oligodendrocytes was not restricted to the ipsilateral hemisphere but was also present in the contralateral striatum (Figures 1A,D,E). This suggests that CBD-tau can induce transmittable changes in oligodendrocyte population across hemispheres.

Due to anatomical connections of striatal white matter tracts to the corpus callosum, we also analyzed CP13^+^ oligodendrocytes in the corpus callosum. After 1 month of the control-tau homogenate inoculation, approximately one third of oligodendrocytes were CP13^+^ (Figure 1A). At that time point, no significant differences in CP13^+^ oligodendrocytes were observed between control-tau and CBD-tau injected hTau animals in either the contra- or ipsilateral sides (Figures 1F,G, respectively). Although in line with striatal results, 12 months after homogenate inoculation, CP13^+^ oligodendrocytes were significantly more frequently observed in CBD-tau than in control-tau injected hTau animals (Figure 1A) in both the contra- and ipsilateral sides (Figures 1H,I, respectively). This confirms that tau-enriched brain homogenate from a CBD case induced transferrable changes in the oligodendrocyte population also in the corpus callosum. Furthermore, the significant positive correlation in the fraction of CP13^+^ oligodendrocytes was observed between the striatum and corpus callosum (Figure 1J), suggesting the presence of the oligodendrocytes' transmissible changes in tau phosphorylation between these structures. Interestingly, when comparing the spread of Ser202 phosporylated tau pathology across hemispheres, the significant positive correlation between the number of ipsi- and contralateral CP13^+^ oligodendrocytes was present only in CBD-tau (Figure 1K) and not control-tau (Figure 1L) injected animals. This supports that only the CBD-tau, not control-tau, is able to trigger and transmit pathology across hemispheres.

**Figure 2:**
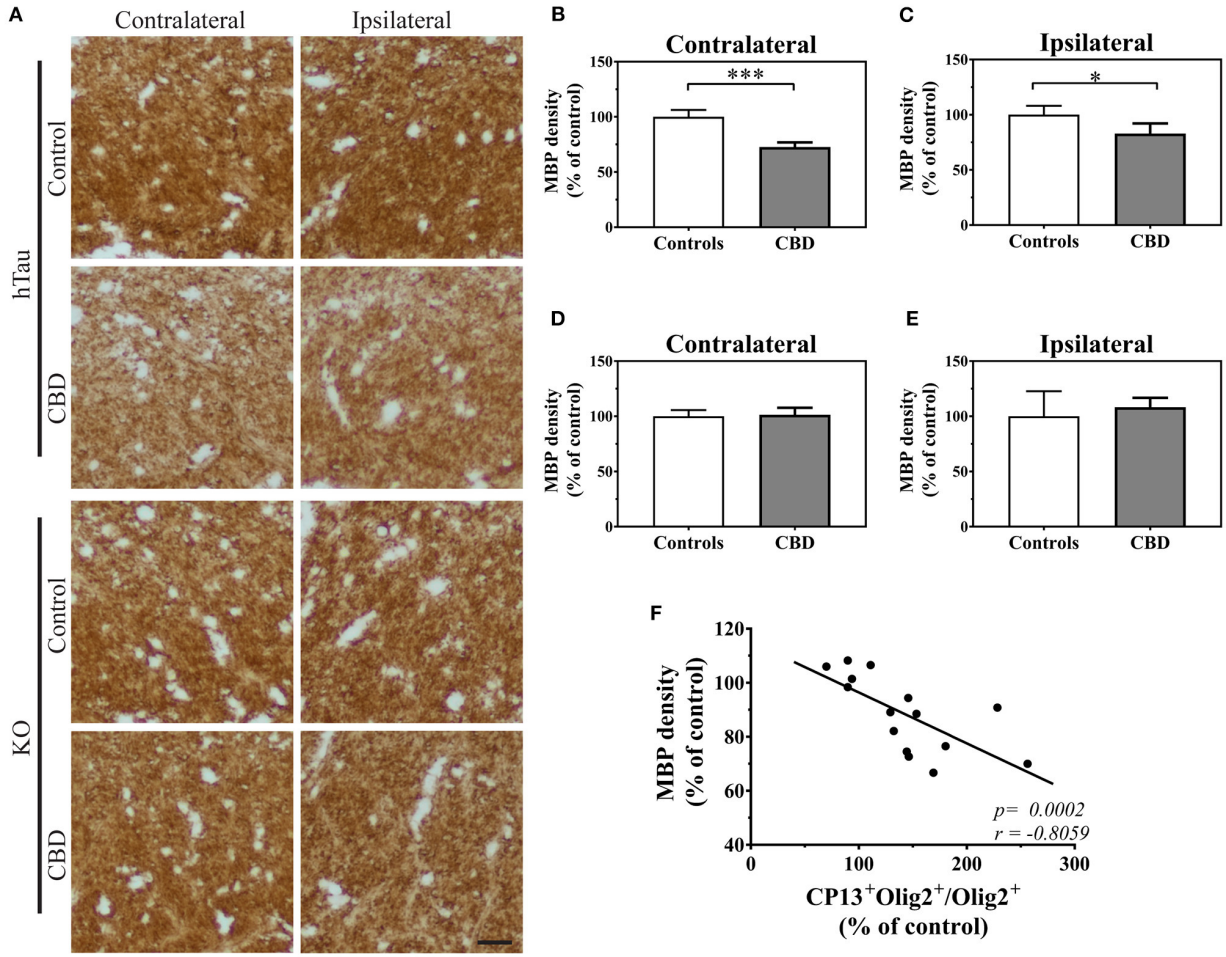
The functional changes in oligodendrocytes triggered by CBD-tau inoculation. **(A)**, the corpus callosum microphotography pictures of myelin basic protein (MBP) immunoreactivity in the contralateral (left panel) and ipsilateral (right panel) sides in hTau (upper panel) or KO (lower panel) animals 12 months after control-tau (upper subpanel) or CBD-tau (lower sub panel) injection. The scale bar represents 25 μm and applies to all pictures. **(B–E)**, The quantitative analysis of densitometric data of MBP immunoreactivity in the corpus callosum of hTau animals contralateral (**B**, controls *n* = 4, CBD *n* = 4) and ipsilateral (**C**, controls *n* = 4, CBD *n* = 4) hemispheres; and KO animals contralateral (**D**, controls *n* = 3, CBD *n* = 4) and ipsilateral (**E**, controls *n* = 3, CBD *n* = 4) hemispheres. The significant decrease in MBP densitometry was observed bilaterally only in hTau animals, reflecting presence of tau-dependent changes. **(F)**, correlation of MBP density and double-positive oligodendrocytes in the corpus callosum. The presence of significant positive correlation indicates functional changes in oligodendrocytes upon tau pathology presence. Comparison between two groups was performed using the Student *t* test. Spearman's rank correlation coefficient was used to analyze dependence between two sets of data. *P*-value < 0.05 was considered statistically significant. *P < 0.05, ***P < 0.0001.

Subsequently, we studied if an increased fraction of CP13^+^ oligodendrocytes could be reflected in their functional changes. To assess this possibility, we measured myelin basic protein (MBP) immunoreactivity in the corpus callosum of 12-month post-injected hTau and KO animals. The densitometric analysis revealed a significant decrease in MBP immunoreactivity in CBD-tau compared with control-tau injected hTau animals (Figures 2A–C). On the contrary, analysis of tau KO animals did not show any significant differences in MBP density between CBD-tau and control-tau injected animals (Figures 2A,D,E), highlighting the importance of human endogenous tau presence in triggering CBD-tau pathology. In addition, the significant negative correlation was detected between MBP density and the number of CP13^+^ oligodendrocytes in the corpus callosum of hTau mice (Figure 2F), indicating the myelin disruption as a functional consequence of increased Ser202 hyperphosphorylated tau in oligodendrocytes.

**Figure 3:**
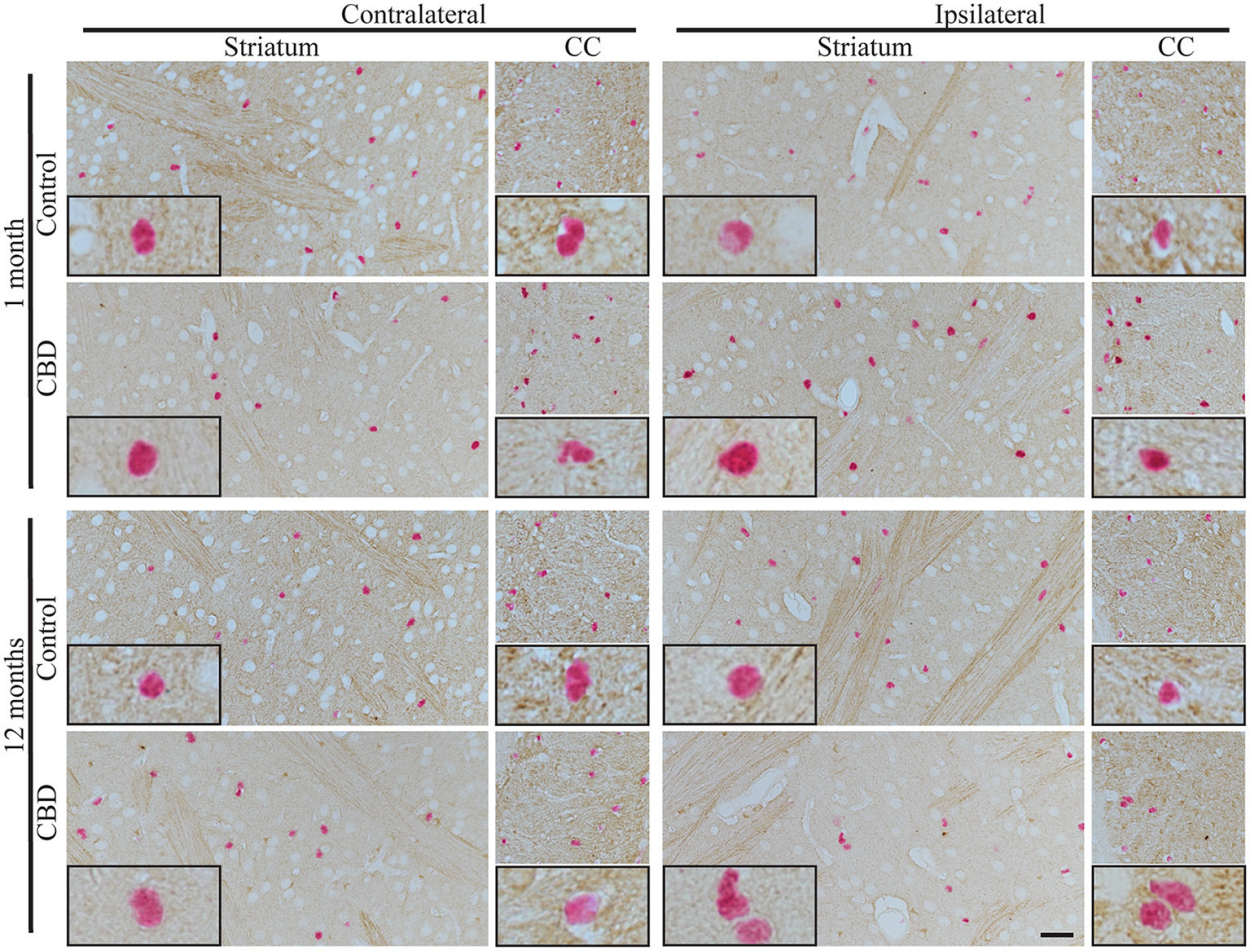
The absence of tau pathology transmission in astrocytes. The microphotography pictures of double immunohistochemistry staining of tau phosphorylated at Ser202 (CP13 antibody, brown chromogen) in astrocytes (Sox9, nuclear marker, red/pink chromogen). The cell distribution in the contralateral (left panel) and ipsilateral side (right panel) of striatum (left subpanel) and corpus callous (right subpanel) after 1 month (upper panel) and 12 months (lower panel) of control-tau (upper subpanel) or CBD-tau (lower sub panel) inoculation. The representative pictures of cells in each group are presented as black-bordered inserts and demonstrate the absence of pathological tau in astrocytes in any of the experimental group at any time points, and similarly, the absence of pathological tau in neurons (morphological cell distinction) in any of the experimental group at any time points. The scale bar represents 50 μm and applies to all pictures; the black-bordered inserts represent 4× digital enlargement.

Because CBD is primarily an astrogliopathy, we analyzed tau pathology in astrocytes. We applied Sox9, a nuclear marker specific for astrocytes, to identify astrocytes in the adult mouse brain outside the neurogenic regions. Examination of striatal astrocytes revealed that CP13^+^ astrocytes are a very rare event in hTau animals, and the vast majority of astrocytes are CP13^−^. Similar results were observed in control-tau and CBD-tau injected animals at both experimental time points (Figure 3). Likewise, the astrocyte population in the corpus callosum also remained mainly CP13^−^ in all experimental groups (Figure 3).

Furthermore, we also analyzed Ser202-specific tau phosphorylation in the neurons of the striatum, and no significant alterations were observed in any of the experimental groups at any of the time points (Figures 1A, 3, based on neuronal morphological discrimination). The corpus callosum was not examined due to a lack of neurons in this structure. The clear neuronal immunoreactivity of CP13 was present in the hippocampus (Supplementary Figure 2) and other brain regions in hTau animals indicating that CP13 antibody recognized pSer202 tau not only in oligodendrocytes, but also neurons (22–24).

**Figure 4:**
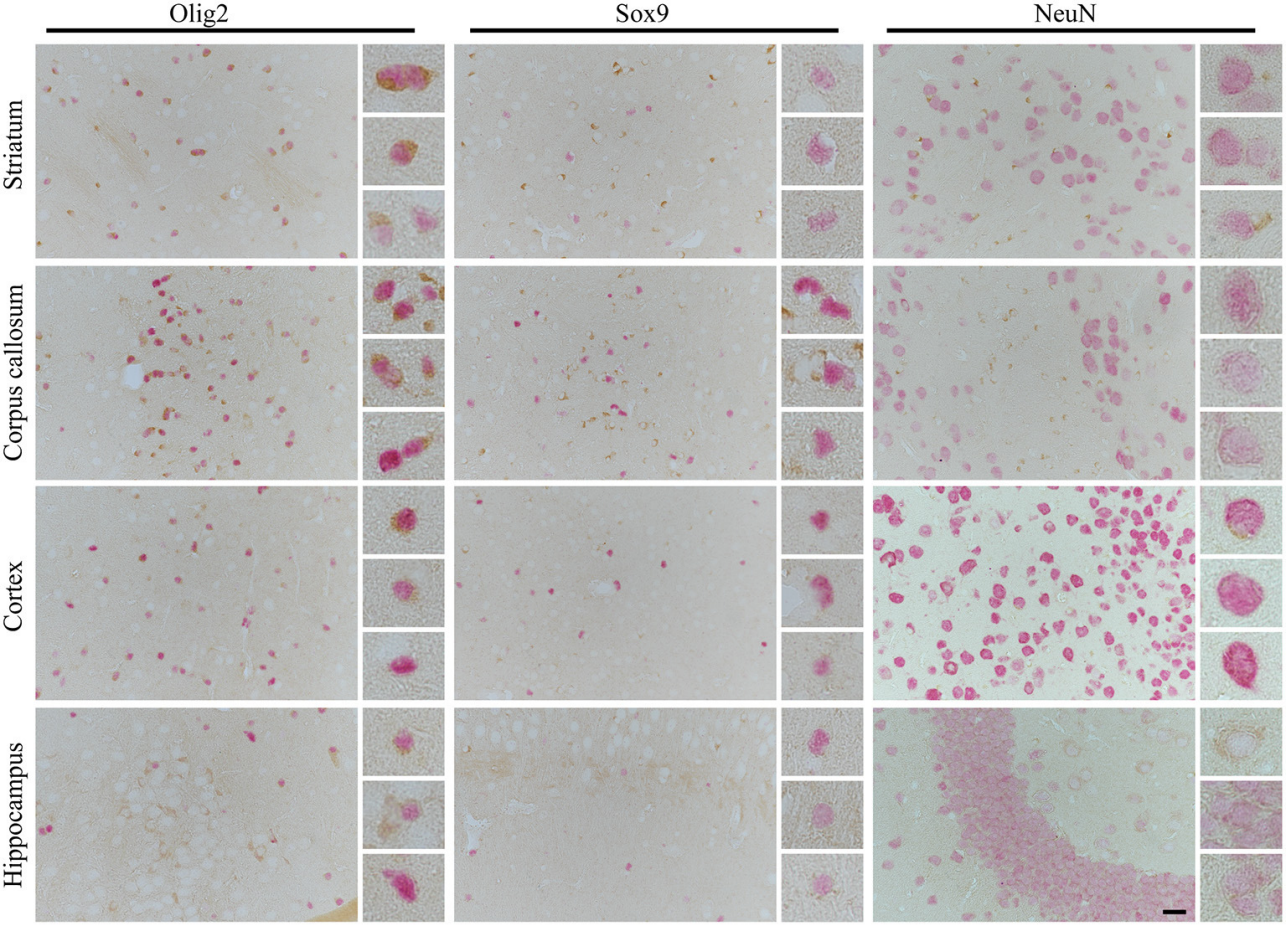
The cell- and side-specific expression of four repeats of tau (4R) isoform in hTau animals brain. The high-power microphotography of double immunohistochemistry staining of 4R tau isoform (RD4, brown chromogen) combined with oligodendrocytes (Olig2, left panel, red/pink chromogen), astrocytic (Sox9, middle panel, red/pink chromogen) or neuronal (NeuN, right panel, red/pink chromogen) markers. The representative pictures of four brain regions: striatum (upper panel), corpus callosum (second upper panel), cortex (third upper panel), and hippocampus (lower panel) with higher magnification of representative cells on the right side of each image. In striatum and corpus callosum only oligodendrocytes were RD4 positive, and no RD4 immunoreactive astrocytes or neurons were detected. In cortex only, some oligodendrocytes and rear neurons were RD4 positive, and no RD4 immunoreactive astrocytes were identified. Similarly, in the hippocampus, no astrocytes were RD4 positive, and only some oligodendrocytes and neurons were weakly RD4 immunoreactive. The scale bar represents 25 μm and applies to all pictures; inserts represent 4× digital enlargement.

Finally, to determine the possible cause of preferential oligodendrocyte susceptibility to CBD-tau induced changes over other cell types in the striatum and corpus callosum, we performed 4R tau-specific immunorectivity to follow subclassification of CBD as a 4R tauopathies (Figure 4). Interestingly, we identified that the only 4R positive cells in the striatum and corpus callosum were oligodendrocytes, and no 4R tau-positive astrocytes or neurons were observed. Furthermore, none or weak expression of 4R tau were detected in oligodendrocytes in the cortex or hippocampus. Only a few 4R tau-positive neurons were observed in the cortex and some in the hippocampus. In none of the analyzed structures were 4R tau positive astrocytes identified. Interestingly, this suggests that 4R tau expression in hTau animals is both cell and site specific.

## Discussion

Numerous studies have been performed with intracerebral injection of human brain homogenates from a range of tauopathies in mice expressing human tau (19, 25–29) or WT animals (29–33). In the present study, by applying a humanized tau (hTau) mouse line (22), we generated a new model in which CBD-tau transmission could be examined not only in the presence of all six human tau isoforms, but also excluding any interaction resulting from endogenous murine tau. The important advantage of the applied model is a non-predominant 4R tau environment, which allows the 3R and 4R tau isoforms distinguishable study. Additionally, tau overexpression in this line is driven by the endogenous tau promoter. Furthermore, performed striatal inoculation relates to the brain region mostly affected by astrogliopathy in the early stages of CBD (7). In our research, the manifestation of pathological tau was analyzed by monitoring the presence of phosphoepitope Ser202 on tau protein by CP13 antibody immunoreactivity, commonly used to detect tau pathology in both early (pretangle) and more advanced stages of neurofibrillary tangle accumulation (22–24).

We show that CBD-tau brain homogenate inoculation in striatum of hTau animals caused hyperphosphorylation of tau at Ser202 predominantly in oligodendrocytes. Next, the pathology of tau hyperphosphorylated at Ser202 was transmitted from the striatum to the overlaying corpus callosum in the ipsilateral side and 12 months postinjection was also detected in oligodendrocytes in the contralateral corpus callosum and striatum. These results are in line with well-documented oligodendrocytes and white matter pathology in CBD patients (4–6, 8, 9). Although astrogliopathy predominates at the earliest stage of CBD pathology, the oligodendroglia involvement in the early preclinical stages is also reported in patients (7). Interestingly, the striatum (caudate and putamen) was the most affected by astrocytic plaque in the earliest stages, and astrogliopathy was accompanied by oligodendroglia pathology present in form of coiled bodies in the putamen (7).

Furthermore, the results showing oligodendrocyte involvement in CBD-tau propagation in our model are in line with previously reported studies in WT (31) and other transgenic mice (26, 27, 29, 34). These studies report oligodendrocytic next to neuronal and astrocytic involvement in CBD-tau propagation although with discrepancy in the cell types involved and timing in both WT and transgenic animals. Interestingly, intracerebral inoculation of the same tauopathies' brain homogenates in the same mouse brain region, presented the diverse magnitude of the cell type involvement in tau spreading depending on whether the animals were WT or transgenic (PS19) (26). In the PS19 transgenic mouse line, expression of the T34 isoform of tau (4R1N) containing the P301S tau mutation is driven by the murine prion protein promoter (35). It is, therefore, not surprising to observe disease pathology in all reported cell types (neurons, astrocytes, and oligodendrocytes) because they have been preconditioned and unified toward it not only by mutation *per se* but also 4R tau isoform presence (both human and endogenous murine). However, even in this preconditioned environment, predominantly oligodendrocytes and white matter pathology was observed, over infrequent intraneuronal tau aggregates upon CBD-tau inoculation for 1 month. Astrocytic pathology was scarcely detected later in time, 6 months postinjection (26). This agrees with our current report, in which exclusive oligodendrocyte involvement over neuronal and astrocytic is observed after striatal CBD-tau inoculation in hTau mice. A non-significant increase in CP13^+^ oligodendrocytes was also detected as early as after 1 month CBD-tau inoculation, and it became significant at 12 months. We did not observe any significant neuronal or astrocytic involvement. However, it should be noted that robust acceleration of pathological changes caused by P301S mutation reported by Lee, Trojanowki, and colleagues (35) and other studies (23, 36–38), together with the tau isoform expression pattern, need to be taken into consideration. On the contrary, in the other study reported by Lee, Trojanowki, and colleagues, CBD-tau injection into WT mice triggered tau aggregates in oligodendrocytes and astrocytes simultaneously 1 month post-injection with accompanying systematic neuronal inclusions (31). In addition, similarly to our study, propagation of oligodendrocyte aggregates was shown over time from the ipsi- to contralateral side of the white matter tracks (fimbria and corpus callosum). Importantly, with our quantitative data, we reported a significant positive correlation between the number of ipsi- and contralateral CP13^+^ oligodendrocytes only in CBD-tau and not control-tau injected animals, reinforcing that only the CBD-tau was able to trigger and transmit pathology across hemispheres.

Likewise, CBD-tau inoculation in ALZ17 animals (27), expressing the longest human tau isoform (4R2N) under the neuron-specific promotor (39) also caused oligodendrocytic tau pathology. The presence of small neurofibrillary tangles and abundant neuropil threads in the hippocampus were observed 6 months after post-hippocampal CBD-tau injection, and were accompanied by tau inclusions in oligodendrocytes in the form of coiled bodies. In line with the aforementioned studies (31, 35) the astrocytic plaques were identified later in time (after 12 months inoculation).

The observed changes in diverse magnitude of the cell type involvement and timing in tau spreading, reported in our and previous studies (26, 27, 31, 34), might be explained by heterologous promoters controlling overexpression of different tau variants (normal or mutated), use of transgenic or WT animals, as well as differences in tau isoform expression. The ratio of 3R and 4R tau isoforms in the human adult brain is 1:1, and adult mice express exclusively 4R isoforms (12, 22, 23, 40–42). The radical changes in tau isoform expression were reported in the literature upon introduction of all six human tau isoforms in a mouse null background (with endogenous tau knockout) animal lines (22, 41), and the predominant expression of 3R over 4R human tau isoforms is observed. Furthermore, as in other mouse lines with null background expressing all six human tau isoforms (hT-PAC-N, Mapt^−^/^−^), we observed indications of the region- and cell-specific tau isoform expression in hTau animals. We found that 4R tau isoforms are only expressed in oligodendrocytes in the striatum and corpus callosum. Furthermore, none or weak expression of 4R tau isoforms were observed in oligodendrocytes in the cortex or hippocampus. No astrocytes in any of the analyzed structures were 4R immunoreactive, and only a few 4R tau positive neurons were detected in cortex, some in the hippocampus, and none were identified in the striatum. This supports our observation that CBD, as a predominantly 4R tau disorder, triggered tau pathology mainly in oligodendrocytes, the only striatal cells in hTau animals expressing 4R tau. In addition, we show that disease transmission is triggered in anatomically connected regions only in the cells expressing 4R tau isoforms. This greatly correlates with a previously published *in vitro* study in which tau fibrils from different tauopathies recruited tau isoforms corresponding to the original human cases (4R recruits 4R, 3R+4R recruits 3R and 4R) (31). Likewise, a recent study using a newly generated 6hTau transgenic line (expressing an equal ratio of 3R and 4R human tau isoforms in null-background mice) states that distinct tauopathy strains recruit the corresponding tau isoforms present in AD, PSP, CBD, and PiD into tau aggregates in 6hTau mice (34). Importantly, in line with our report, the cross-seeding of non-corresponding tau isoforms was inefficient in PSP and CBD in contrary to AD. Moreover, oligodendrocytes together with neuronal and astrocyte tau pathologies was observed upon CBD-tau inoculation.

Furthermore, a recent study on human tau pathology transition by glia in the absence of neuronal tau supports our observation (29). In the absence of neuronal tau pathology (no neuronal tau expression), the propagation of CBD-tau pathology across the mouse brain was observed only in oligodendroglia in the fimbria and corpus callosum, which agrees with our report. In addition, astrocytic tau aggregates did not spread in CBD-tau injected animals in the absence of neuronal tau, and neuronal tau aggregate release and local uptake by astrocytes was suggested as a basis of astrogliopathy. Furthermore, in this study reported by Lee, Trojanowki, and colleagues (29) the oligodendrocyte loss is reported, and “oligodendroglial connectome” is suggested as a tau propagation road. Our data also suggest oligodendrocyte connectome-based tau transmission. The oligodendrocyte connectome is based on the gap junctions (GJ) formed by connexin and pannexin transmebrane proteins (43–45). The GJ allows exchange of small molecules, ions, and metabolites between coupled cells. The connexin-43 (Cx42), as reported by the Lee and Trojanowki team, is highlighted together with aquaporin-4 as markers of aging-related tau astrogliopathy (46). Although this study is connecting tau pathology with connexin, highly expressed in astrocytes and generating homotypic channels (Cx42/Cx42) in astrocyte–astrocyte coupling, Cx42 is also highly involved in generating heterotypic channels in astrocyte–oligodendrocyte coupling with connexin-32 (Cx32). The Cx32 is highly expressed by oligodendrocytes and recently reported to be pivotal in α-synuclein oligomer uptake and transfer in neurons and oligodendrocytes (47). Furthermore, a number of small compounds and ions that alter tau aggregation have been reported (48, 49). For example, the zinc ion (Zn^2+^) is one of the most potent tau aggregation stimulators. Importantly, in mature oligodendrocytes, the myelin sheath integrity was indicated to be greatly dependent on MBP and PLP complexes with Zn^2+^ (50–53). In addition, a Zn^2+^ involvement in pathologies of Alzheimer's, Parkinson's, and other neurodegenerative diseases is reported (51).

Although it did not include CBD, a previous study describing oligodendrocytic involvement in tau seeding and spreading from a variety of tauopathies by white matter injection is in line with our present data (33). The inoculation of sarkosyl-insoluble homogenates into the corpus callosum of WT animals triggered tau pathology in oligodendrocytes without any neuronal and astrocytic pathology. Furthermore, both tau seeding and spreading in oligodendrocytes were active processes with phospho-kinase involvement and, comparable to our data, extending from the ipsi- to contralateral hemisphere. Also, several reports using transgenic mice expressing tau mutations display inclusions in oligodendrocytes in addition to neuronal and astrocytes (54–58). Besides this, the pattern of glial tau pathology spreading in PS19 animals upon CBD-tau inoculation differed dramatically from intra-axonal spreading and the occurrence of the axonal afferent- and efferent-independent pattern has been proposed, which is consistent with our report (26).

Importantly, in the current study, we also report the functional consequences of increased oligodendrocytes with tau hyperphosphorylated at Ser202. We show the significant decrease in MBP density in the corpus callosum, both ipsi- and contralaterally, in 12 months CBD-tau inoculated hTau animals in comparison to controls. Furthermore, reduction in MBP density was not observed in tau KO mice injected either with CBD-tau or control-tau. This highlights the importance of an endogenous tau presence in CBD-tau pathology transmission and triggering of the subsequent functional changes. In addition, the significant negative correlation was detected between MBP density and number of CP13^+^ oligodendrocytes in corpus callosum, further strengthening the myelin disruption as a functional consequence of increased Ser202 hyperphosphorylated tau in oligodendrocytes. This is in line with the aforementioned well-documented oligodendrocytic and white matter pathology in CBD patients (8, 9). It is also interesting to note that large-scale genetic studies have reported that gene variants in myelin-associated oligodendrocyte basic protein confers risk to develop CBD and PSP (59, 60).

Our research supports the emerging hypothesis that the accumulation of tau in oligodendrocytes might cause neurodegeneration by disrupting axonal transport (36). In this context, it is important to mention that oligodendrocytes are the most numerous glia cells in the brain (61). Moreover, their classical role, myelin-based electric insulation to axons, in optimizing axon potential conduction has been extended to providing a trophic support to long axons (62, 63), white matter angiogenesis (64), and increasing tightness of the blood–brain barrier (65). To our knowledge, we are the first reporting that non-mutated human tau could contribute to MBP dysfunction upon CBD-tau inoculation. The previous study presents structural disruption and progressive loss of myelin in T34 P301S mutant mice, but not in T34 tau overexpressing oligodendrocytes in CNP promotor-driven (oligodendrocyte-specific) transgenic animals (36). In this study, impairment in axonal transport preceded axonal degeneration and correlates with early stages of tau aggregate accumulation in oligodendrocytes. In another report, the signs of slightly disrupted myelin are reported in corpus callosum–injected WT animals by a variety of tauopathies in diverse magnitudes by presenting proteolipid protein 1 immunoreactive balls and dots (although this study did not include any CBD cases) (33).

In conclusion, we demonstrate new insights into cell and regional selectivity of tau spreading in sporadic CBD tauopathy. We show that CBD-tau brain homogenate inocculation in the striatum of hTau animals caused hyperphosphorylation of tau at Ser202, predominantly in oligodendrocytes. Next, the pathology was transmitted from striatum to overlaying corpus callosum in the ipsilateral side and 12 months postinjection also in oligodendrocytes in the contralateral corpus callosum and striatum. Moreover, we report that the almost exclusive oligodendrocyte-based transmission of hyperphosphorylated tau is reflected by the endogenous 4R tau isoforms immunoreactivity. Furthermore, we demonstrate that non-mutated human tau could contribute to myelin dysfunction, reflected by decreased MBP immunoreactivity, upon CBD-tau inoculation as the functional consequence of increased oligodendrocytes with tau hyperphosphorylated at Ser202.

The question of why tauopathies vary greatly not only in histopathological but also neuroanatomical patterns could be partially answered by region- and cell-specific tau isoform presence in the human brain; however, additional larger studies with more CBD cases are required to elucidate this issue.

## Data Availability Statement

The raw data supporting the conclusions of this article will be made available by the authors, without undue reservation.

## Ethics Statement

The animal study was reviewed and approved by Stockholm North Ethical Committee.

## Author Contributions

KP and PS designed research. JZ-P, KP, and LK performed research. JZ-P, TR, and PS analyzed data and JZ-P and PS wrote the paper. All authors contributed to the article and approved the submitted version.

## Conflict of Interest

The authors declare that the research was conducted in the absence of any commercial or financial relationships that could be construed as a potential conflict of interest.

## References

[1] Togasaki DM Tanner CM. Epidemiologic aspects. Adv Neurol. (2000) 82:53–9. 10624470

[2] Schrag A Ben-Shlomo Y Quinn NP. Prevalence of progressive supranuclear palsy and multiple system atrophy: a cross-sectional study. Lancet. (1999) 354:1771–5. 10.1016/S0140-6736(99)04137-9 10577638

[3] Winter Y Bezdolnyy Y Katunina E Avakjan G Reese JP Klotsche J. Incidence of Parkinson's disease and atypical parkinsonism: Russian population-based study. Mov Disord. (2010) 25:349–56. 10.1002/mds.22966 20108378

[4] Dickson DW Neuropathologic differentiation of progressive supranuclear palsy and corticobasal degeneration. J Neurol. (1999) 246:II6–15. 10.1007/BF03161076 10525997

[5] Ikeda K Akiyama H Haga C Kondo H Arima K Oda T. Argyrophilic thread-like structure in corticobasal degeneration and supranuclear palsy. Neurosci Lett. (1994) 174:157–9. 10.1016/0304-3940(94)90010-8 7526285

[6] Arima K Nakamura M Sunohara N Ogawa M Anno M Izumiyama Y. Ultrastructural characterization of the tau-immunoreactive tubules in the oligodendroglial perikarya and their inner loop processes in progressive supranuclear palsy. Acta Neuropathol (Berl). (1997) 93:558–66. 10.1007/s004010050652 9194894

[7] Ling H Kovacs GG Vonsattel JP Davey K Mok KY Hardy J. Astrogliopathy predominates the earliest stage of corticobasal degeneration pathology. Brain. (2016) 139:3237–52. 10.1093/brain/aww256 27797812

[8] Upadhyay N Suppa A Piattella MC Bologna M Di Stasio F Formica A. MRI gray and white matter measures in progressive supranuclear palsy and corticobasal syndrome. J Neurol. (2016) 263:2022–31. 10.1007/s00415-016-8224-y 27411806

[9] Boxer AL Geschwind MD Belfor N Gorno-Tempini ML Schauer GF Miller BL. Patterns of brain atrophy that differentiate corticobasal degeneration syndrome from progressive supranuclear palsy. Arch Neurol. (2006) 63:81–6. 10.1001/archneur.63.1.81 16401739

[10] Spillantini MG Yoshida H Rizzini C Lantos PL Khan N Rossor MN. A novel tau mutation (N296N) in familial dementia with swollen achromatic neurons and corticobasal inclusion bodies. Ann Neurol. (2000) 48:939–43. 10.1002/1531-8249(200012)48:6<939::AID-ANA17>3.0.CO;2-1 11117553

[11] Kahlson MA Colodner KJ. Glial Tau Pathology in Tauopathies: Functional Consequences. J Exp Neurosci. (2015) 9:43–50. 10.4137/JEN.S25515 26884683 PMC4750898

[12] Goedert M Jakes R. Expression of separate isoforms of human tau protein: correlation with the tau pattern in brain and effects on tubulin polymerization. EMBO J. (1990) 9:4225–30. 10.1002/j.1460-2075.1990.tb07870.x 2124967 PMC552204

[13] Dickson DW Kouri N Murray ME Josephs KA. Neuropathology of frontotemporal lobar degeneration-tau (FTLD-tau). J Mol Neurosci. (2011) 45:384–9. 10.1007/s12031-011-9589-0 21720721 PMC3208128

[14] Alonso AD Grundke-Iqbal I Barra HS Iqbal K. Abnormal phosphorylation of tau and the mechanism of Alzheimer neurofibrillary degeneration: sequestration of microtubule-associated proteins 1 and 2 and the disassembly of microtubules by the abnormal tau. Proc Natl Acad Sci USA. (1997) 94:298–303. 10.1073/pnas.94.1.298 8990203 PMC19321

[15] Cleveland DW Hwo SY Kirschner MW. Physical and chemical properties of purified tau factor and the role of tau in microtubule assembly. J Mol Biol. (1977) 116:227–47. 10.1016/0022-2836(77)90214-5 146092

[16] Brunello CA Merezhko M Uronen RL Huttunen HJ. Mechanisms of secretion and spreading of pathological tau protein. Cell Mol Life Sci. (*2*019) 77:1721–44. 10.1007/s00018-019-03349-1 31667556 PMC7190606

[17] Wegmann S Eftekharzadeh B Tepper K Zoltowska KM Bennett RE Dujardin S. Tau protein liquid-liquid phase separation can initiate tau aggregation. EMBO J. (2018) 37:e98049. 10.15252/embj.201798049 29472250 PMC5881631

[18] Qiang L Sun X Austin TO Muralidharan H Jean DC Liu M. Tau does not stabilize axonal microtubules but rather enables them to have long labile domains. Curr Biol. (2018) 28:2181–9.e4. 10.1016/j.cub.2018.05.045 30008334

[19] Clavaguera F Bolmont T Crowther RA Abramowski D Frank S Probst A. Transmission and spreading of tauopathy in transgenic mouse brain. Nat Cell Biol. (2009) 11:909–13. 10.1038/ncb1901 19503072 PMC2726961

[20] Paslawski W Zareba-Paslawska J Zhang X Hölzl K Wadensten H Shariatgorji M. α-synuclein–lipoprotein interactions and elevated ApoE level in cerebrospinal fluid from Parkinson's disease patients. Proc Natl Acad Sci. (2019) 116: 15226–35. 10.1073/pnas.1821409116 31270237 PMC6660770

[21] Abramoff MD Magelhaes PJ Ram SJ Image processing with ImageJ. Biophotonics Int. (2004) 11:36–42.

[22] Andorfer C Kress Y Espinoza M de Silva R Tucker KL Barde YA. Hyperphosphorylation and aggregation of tau in mice expressing normal human tau isoforms. J Neurochem. (2003) 86:582–90. 10.1046/j.1471-4159.2003.01879.x 12859672

[23] Lewis J McGowan E Rockwood J Melrose H Nacharaju P Van Slegtenhorst M. Neurofibrillary tangles, amyotrophy and progressive motor disturbance in mice expressing mutant (P301L) tau protein. Nat Genet. (2000) 25:402–5. 10.1038/78078 10932182

[24] Xia Y Prokop S K. Gorion MM Kim JD Sorrentino ZA Bell BM. Tau Ser208 phosphorylation promotes aggregation and reveals neuropathologic diversity in Alzheimer's disease and other tauopathies. Acta Neuropathol Commun. (2020) 8:88. 10.1186/s40478-020-00967-w 32571418 PMC7310041

[25] Ahmed Z Cooper J Murray TK Garn K McNaughton E Clarke H. A novel in vivo model of tau propagation with rapid and progressive neurofibrillary tangle pathology: the pattern of spread is determined by connectivity, not proximity. Acta Neuropathol. (2014) 127:667–83. 10.1007/s00401-014-1254-6 24531916 PMC4252866

[26] Boluda S Iba M Zhang B Raible KM Lee VM Trojanowski JQ Differential induction and spread of tau pathology in young PS19 tau transgenic mice following intracerebral injections of pathological tau from Alzheimer's disease or corticobasal degeneration brains. Acta Neuropathol. (2015) 129:221–37. 10.1007/s00401-014-1373-0 25534024 PMC4305460

[27] Clavaguera F Akatsu H Fraser G Crowther RA Frank S Hench J. Brain homogenates from human tauopathies induce tau inclusions in mouse brain. Proc Natl Acad Sci USA. (2013) 110:9535–40. 10.1073/pnas.1301175110 23690619 PMC3677441

[28] Hu W Zhang X Tung YC Xie S Liu F Iqbal K. Hyperphosphorylation determines both the spread and the morphology of tau pathology. Alzheimers Demen. (2016) 12:1066–1077. 10.1016/j.jalz.2016.01.014 27133892

[29] Narasimhan S Changolkar L Riddle DM Kats A Stieber A Weitzman SA. Human tau pathology transmits glial tau aggregates in the absence of neuronal tau. J Exp Med. (2020) 217:e20190783. 10.1084/jem.20190783 31826239 PMC7041709

[30] Guo JL Narasimhan S Changolkar L He Z Stieber A Zhang B. Unique pathological tau conformers from Alzheimer's brains transmit tau pathology in nontransgenic mice. J Exp Med. (2016) 213:2635–54. 10.1084/jem.20160833 27810929 PMC5110027

[31] Narasimhan S Guo JL Changolkar L Stieber A McBride JD Silva LV. Pathological tau strains from human brains recapitulate the diversity of tauopathies in nontransgenic mouse brain. J Neurosci Nurs. (2017) 37:11406–23. 10.1523/JNEUROSCI.1230-17.2017 29054878 PMC5700423

[32] Audouard E Houben S Masaracchia C Yilmaz Z Suain V Authelet M. High-molecular-weight paired helical filaments from alzheimer brain induces seeding of wild-type mouse tau into an argyrophilic 4R tau pathology in vivo. Am J Pathol. (2016) 186:2709–22. 10.1016/j.ajpath.2016.06.008 27497324

[33] Ferrer I Aguilo Garcia M Carmona M Andres-Benito P Torrejon-Escribano B Garcia-Esparcia. involvement of oligodendrocytes in tau seeding and spreading in tauopathies. Front Aging Neurosci. (2019) 11:112. 10.3389/fnagi.2019.00112 31191295 PMC6546889

[34] He Z McBride JD Xu H Changolkar L Kim S-j Zhang B. Transmission of tauopathy strains is independent of their isoform composition. Nature Commun. (2020) 11:7. 10.1038/s41467-019-13787-x 31911587 PMC6946697

[35] Yoshiyama Y Higuchi M Zhang B Huang SM Iwata N Saido TC. Synapse loss and microglial activation precede tangles in a P301S tauopathy mouse model. Neuron. (2007) 53:337–51. 10.1016/j.neuron.2007.01.010 17270732

[36] Higuchi M Zhang B Forman MS Yoshiyama Y Trojanowski JQ Lee VM. Axonal degeneration induced by targeted expression of mutant human tau in oligodendrocytes of transgenic mice that model glial tauopathies. J Neurosci. (2005) 25:9434–43. 10.1523/JNEUROSCI.2691-05.2005 16221853 PMC6725712

[37] Tanemura K Murayama M Akagi T Hashikawa T Tominaga T Ichikawa M. Neurodegeneration with tau accumulation in a transgenic mouse expressing V337M human tau. J Neurosci. (2002) 22:133–41. 10.1523/JNEUROSCI.22-01-00133.2002 11756496 PMC6757582

[38] Zhang B Higuchi M Yoshiyama Y Ishihara T Forman MS Martinez D. Retarded axonal transport of R406W mutant tau in transgenic mice with a neurodegenerative tauopathy. J Neurosci. (2004) 24:4657–67. 10.1523/JNEUROSCI.0797-04.2004 15140937 PMC6729383

[39] Probst A Götz J Wiederhold KH Tolnay M Mistl C Jaton AL. Axonopathy and amyotrophy in mice transgenic for human four-repeat tau protein. Acta Neuropathol. (2000) 99:469–81. 10.1007/s004010051148 10805089

[40] Goedert M Spillantini MG Jakes R Rutherford D Crowther RA. Multiple isoforms of human microtubule-associated protein tau: sequences and localization in neurofibrillary tangles of Alzheimer's disease. Neuron. (1989) 3:519–26. 10.1016/0896-6273(89)90210-9 2484340

[41] McMillan P Korvatska E Poorkaj P Evstafjeva Z Robinson L Greenup L. Tau isoform regulation is region- and cell-specific in mouse brain. J Comp Neurol. (2008) 511:788–803. 10.1002/cne.21867 18925637 PMC2845852

[42] Goedert M Spillantini MG Potier MC Ulrich J Crowther RA. Cloning and sequencing of the cDNA encoding an isoform of microtubule-associated protein tau containing four tandem repeats: differential expression of tau protein mRNAs in human brain. EMBO J. (1989) 8:393–9. 10.1002/j.1460-2075.1989.tb03390.x 2498079 PMC400819

[43] Nualart-Marti A Solsona C Fields RD. Gap junction communication in myelinating glia. Biochim Biophys Acta. (2013) 1828:69–78. 10.1016/j.bbamem.2012.01.024 22326946 PMC4474145

[44] Vejar S Oyarzún JE Retamal MA Ortiz FC Orellana JA. Connexin and pannexin-based channels in oligodendrocytes: implications in brain health and disease. Front Cell Neurosci. (2019) 13:3. 10.3389/fncel.2019.00003 30760982 PMC6361860

[45] Simons M Trajkovic K. Neuron-glia communication in the control of oligodendrocyte function and myelin biogenesis. J Cell Sci. (2006) 119:4381. 10.1242/jcs.03242 17074832

[46] Kovacs GG Yousef A Kaindl S Lee VM Trojanowski JQ. Connexin-43 and aquaporin-4 are markers of ageing-related tau astrogliopathy (ARTAG)-related astroglial response. Neuropathol Appl Neurobiol. (2018) 44:491–505. 10.1111/nan.12427 28755467 PMC5788733

[47] Reyes JF Sackmann C Hoffmann A Svenningsson P Winkler J Ingelsson M. Binding of α-synuclein oligomers to Cx32 facilitates protein uptake and transfer in neurons and oligodendrocytes. Acta Neuropathol. (2019) 138:23–47. 10.1007/s00401-019-02007-x 30976973 PMC6570706

[48] Kim AC Lim S Kim YK. Metal ion effects on Aβ and tau aggregation. Int J Mol Sci. (2018) 19:128. 10.3390/ijms19010128 29301328 PMC5796077

[49] Lim S Haque MM Kim D Kim DJ Kim YK. Cell-based models to investigate tau aggregation. Comput Struct Biotechnol J. (2014) 12:7–13. 10.1016/j.csbj.2014.09.011 25505502 PMC4262059

[50] Earl C Chantry A Mohammad N Glynn P. Zinc ions stabilise the association of basic protein with brain myelin membranes. J Neurochem. (1988) 51:718–24. 10.1111/j.1471-4159.1988.tb01803.x 2457650

[51] Mocchegiani E Bertoni-Freddari C Marcellini F Malavolta M. Brain, aging and neurodegeneration: role of zinc ion availability. Prog Neurobiol. (2005) 75:367–90. 10.1016/j.pneurobio.2005.04.005 15927345

[52] Baran C Smith GS Bamm VV Harauz G Lee JS. Divalent cations induce a compaction of intrinsically disordered myelin basic protein. Biochem Biophys Res Commun. (2010) 391:224–9. 10.1016/j.bbrc.2009.11.036 19903451

[53] Lehotzky A Oláh J Szunyogh S Szabó A Berki T Ovádi J. Zinc-induced structural changes of the disordered tppp/p25 inhibits its degradation by the proteasome. Biochim Biophys Acta. (2015) 1852:83–91. 10.1016/j.bbadis.2014.10.015 25445539

[54] Gotz J Tolnay M Barmettler R Chen F Probst A Nitsch RM. Oligodendroglial tau filament formation in transgenic mice expressing G272V tau. Eur J Neurosci. (2001) 13:2131–40. 10.1046/j.0953-816x.2001.01604.x 11422454

[55] Lin W-L Lewis J Yen S-H Hutton M Dickson DW. Filamentous tau in oligodendrocytes and astrocytes of transgenic mice expressing the human tau isoform with the P301L mutation. Am J Pathol. (2003) 162:213–8. 10.1016/S0002-9440(10)63812-6 12507904 PMC1851123

[56] Lin WL Zehr C Lewis J Hutton M Yen SH Dickson DW. Progressive white matter pathology in the spinal cord of transgenic mice expressing mutant (P301L) human tau. J Neurocytol. (2005) 34:397–410. 10.1007/s11068-006-8726-0 16902761

[57] Ren Y Lin WL Sanchez L Ceballos C Polydoro M Spires-Jones TL. Endogenous tau aggregates in oligodendrocytes of rTg4510 mice induced by human P301L tau. J Alzheimers Dis. (2014) 38:589–600. 10.3233/JAD-130986 24028867

[58] Ferrer I. Oligodendrogliopathy in neurodegenerative diseases with abnormal protein aggregates: the forgotten partner. Prog Neurobiol. (2018) 169:24–54. 10.1016/j.pneurobio.2018.07.004 30077775

[59] Hoglinger GU Melhem NM Dickson DW Sleiman PM Wang LS Klei L. Identification of common variants influencing risk of the tauopathy progressive supranuclear palsy. Nat Genet. (2011) 43:699–705. 10.1038/ng.859 21685912 PMC3125476

[60] Kouri N Ross OA Dombroski B Younkin CS Serie DJ Soto-Ortolaza A. Genome-wide association study of corticobasal degeneration identifies risk variants shared with progressive supranuclear palsy. Nature Commun. (2015) 6:7247. 10.1038/ncomms8247 26077951 PMC4469997

[61] Valerio-Gomes B Guimaraes DM Szczupak D Lent R. The absolute number of oligodendrocytes in the adult mouse brain. Front Neuroanat. (2018) 12:90. 10.3389/fnana.2018.00090 30425626 PMC6218541

[62] Wilkins A Chandran S Compston A. A role for oligodendrocyte-derived IGF-1 in trophic support of cortical neurons. Glia. (2001) 36:48–57. 10.1002/glia.1094 11571783

[63] Nave KA. Myelination and the trophic support of long axons. Nat Rev Neurosci. (2010) 11: 275–83. 10.1038/nrn2797 20216548

[64] Yuen TJ Silbereis JC Griveau A Chang SM Daneman R Fancy SPJ. Oligodendrocyte-encoded HIF function couples postnatal myelination and white matter angiogenesis. Cell. (2014) 158:383–96. 10.1016/j.cell.2014.04.052 25018103 PMC4149873

[65] Seo JH Maki T Maeda M Miyamoto N Liang AC Hayakawa K. Oligodendrocyte precursor cells support blood-brain barrier integrity via TGF-beta signaling. PloS One. (2014) 9:e103174. 10.1371/journal.pone.0103174 25078775 PMC4117639

