# Supplementary material for: Tau Isoform-Driven CBD Pathology Transmission in Oligodendrocytes in Humanized Tau Mice
Source: Front Neurol. 2021 Jan 15;11:589471. doi: 10.3389/fneur.2020.589471 (PMC7845573; doi:10.3389/fneur.2020.589471)
Supplement: Supplementary file 1 [file Data_Sheet_1.pdf]

Supplementary Material

1 Supplementary Data

The clinical autopsy protocols of human cases used in study

Control case

Histological examination demonstrated mild small vessel disease largely affecting the putamen. In this control case, there was mild Alzheimer-type pathology: Braak and Braak stage II neurofibrillary tangle pathology in medial temporal lobe structures, Thal phase 3 amyloid pathology with amyloid plaques being present in the neocortex, hippocampal formation, striatum and CERAD sparse neuritic plaques. There were only rare neurofibrillary tangles in the temporal neocortex. Using the National Institute on Aging–Alzheimer’s Association guidelines, these pathological changes corresponded to ‘low’ Alzheimer’s disease neuropathological change (ABC score: A2, B1, C1). In addition, there was TDP43 pathology, which was confined to limbic structures (TDP43 proteinopathy).

CBD case

The frontal cortex was severely affected by nerve cell loss and microvacuolation in the superficial cortical laminae. There were also ballooned, achromatic neurons. AT8 immunohistochemistry highlighted numerous pretangles, scattered neurofibrillary tangles, numerous tau-positive neuropil threads and typical astrocytic plaques. There were numerous neuropil threads and scattered oligodendroglial coiled bodies in the subcortical white matter. In addition to the corticobasal degeneration pathology, there were Alzheimer’s disease neuropathological changes in this case. The Alzheimer’s neurofibrillary tangle pathology corresponded to Braak and Braak stage II, the amyloid pathology to Thal phase 2 and the neuritic plaques were CERAD sparse. Using the National Institute on Aging-Alzheimer’s Association guidelines, the Alzheimer pathology corresponded to ‘low’ Alzheimer's disease neuropathological change (ABC score: A1, B1, C1) (Montine et al. Acta Neuropathol 2012; 123:1-11). There were no additional α-synuclein or TDP43 pathologies.

2 Supplementary Figures

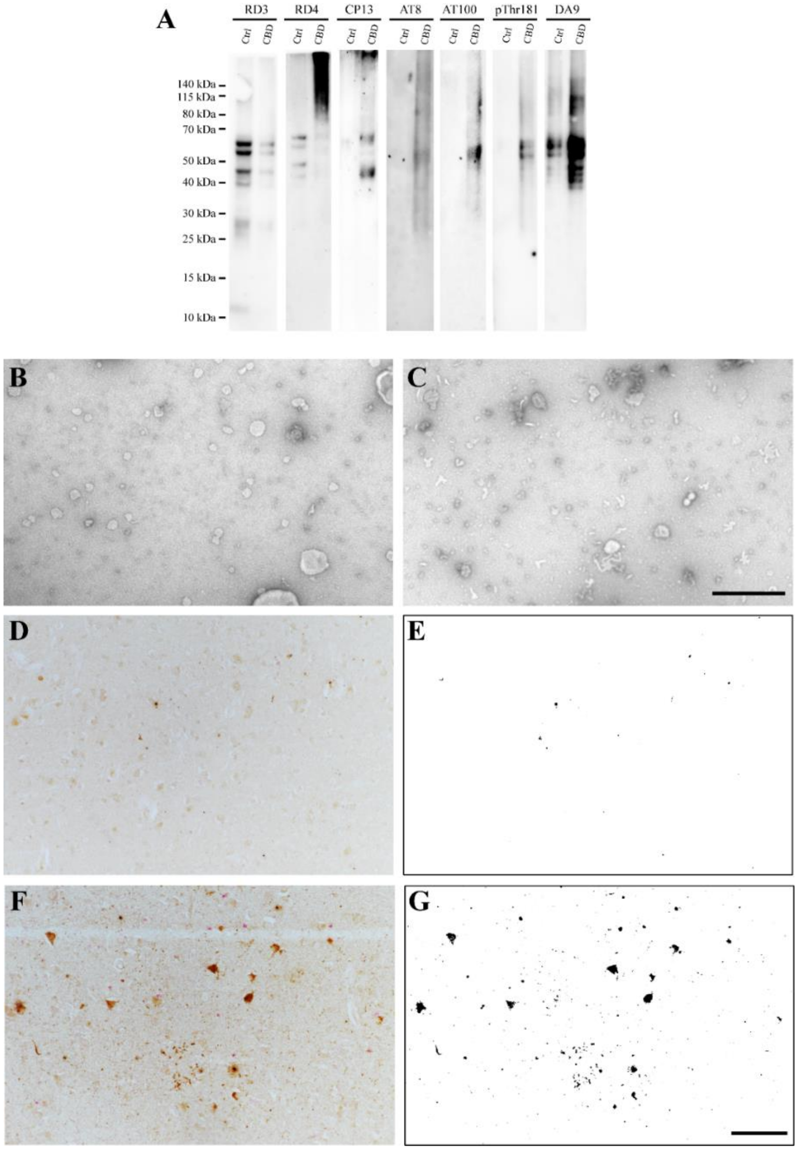

Supplementary Figure 1. Human cases characteristic. A, The tau insoluble brain homogenate fraction analysis with following antibodies RD3 (3R tau isoforms), RD4 (4R tau isoforms), phospho-tau specific antibodies CP13 (Ser2020), AT8 (Ser202, Thr205), AT100 (Thr212, Ser214), pThr181, and DA9 for total tau. **B-C,** negative stain TEM picture of tau insoluble brain homogenate fraction from control (**B**) and CBD case (**C**). The scale bar on C represents 500 nm and applies to B and C. **D,** the control case microphotography pictures of double immunohistochemistry staining of tau phosphorylated at Serine202 (CP13 antibody, brown chromogen) with oligodendrocytes marker (Olig2, nuclear marker, red/pink chromogen). **E,** the negative projection of CP13 immunoreactivity of control case. **F,** the control case microphotography pictures of double immunohistochemistry staining of tau phosphorylated at Serine202 (CP13 antibody, brown chromogen) with oligodendrocytes marker (Olig2, nuclear marker, red/pink chromogen). **G,** the negative projection of CP13 immunoreactivity of CBD case. The scale bar on G represents 100 μm and applies to D-G pictures.

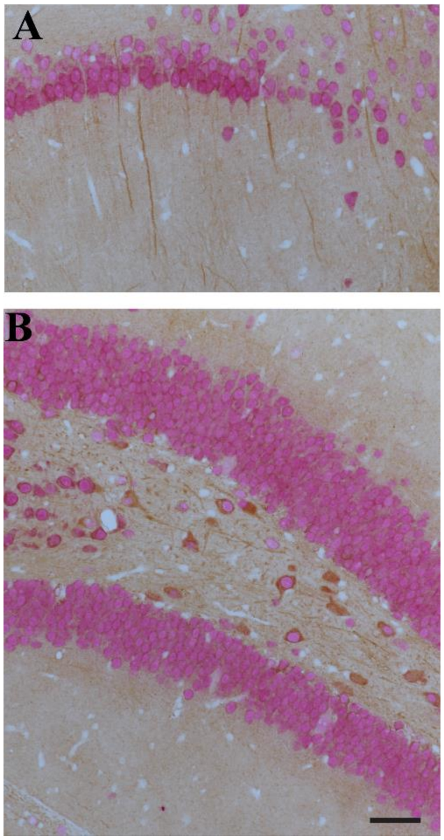

Supplementary Figure 2. The hippocampal immunohistochemistry staining of tau phosphorylated at Ser202 (CP13 antibody) in neurons. The microphotography pictures of double immunohistochemistry staining of tau phosphorylated at Ser202 (CP13 antibody, brown chromogen) in neurons (NeuN, nuclear marker, red/pink chromogen) in CA1 region (**A)** and mossy cells (**B**) of hippocampus. The predominant axonal staining of Ser202 is observed in CA1 region while cell body and axons are clearly stained in mossy cells. The scale bar represents 25 µm and applies to all pictures.
